# New surgical technique for scleral fixation: A novel sutured approach for Carlevale lens implantation

**DOI:** 10.1016/j.ajoc.2025.102343

**Published:** 2025-05-06

**Authors:** Christopher Stewart, Jaskaran Singh-Bhangu, Sushrutha Dissanayake

**Affiliations:** a Faculty of Medicine, Health and Life Science, Institute of Life Science 2, Swansea University, Swansea, UK; b Department of Ophthalmology, Singleton Hospital, Swansea Bay University Health Board, Swansea, UK

## Video related to this article

The following is the video related to this article Video 1

Video 1
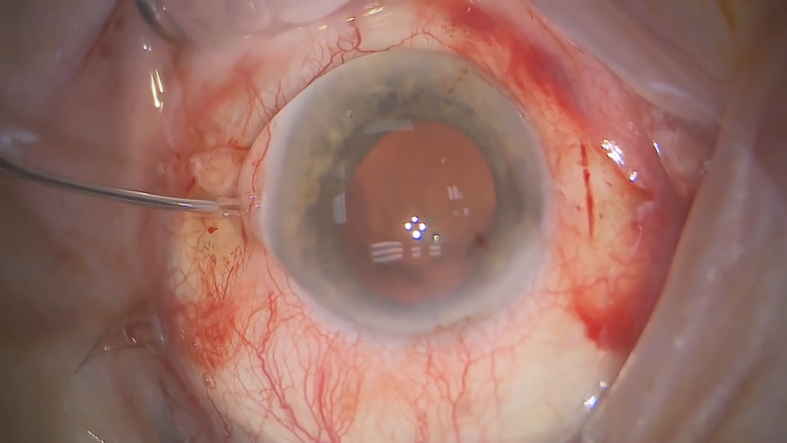

## CRediT authorship contribution statement

**Christopher Stewart:** Writing – review & editing, Writing – original draft, Visualization, Software. **Jaskaran Singh-Bhangu:** Writing – review & editing. **Sushrutha Dissanayake:** Writing – review & editing, Writing – original draft, Data curation, Conceptualization.

## Transcript

This video demonstrates a novel technique for fixing a Carlevale lens to the sclera. The operating surgeon and creator of this technique is Mr Sushrutha Dissanayake. Surgery begins with limited peritomy and haemostasis followed by partial thickness sclerotomies at 3 and 9. This is made using the No. 11 scalpel blade, at three and nine o'clock, at 1.5mm from the limbus. The incision is parallel to a tangent drawn at the limbus and about 3–4 mm in length. At the midpoint of the incision, sclerotomy is done to enter the vitreous cavity using a 23-gauge MVR knife. The lens is then injected via Acujet injector using a 2.75mm keratome (enlarged as necessary), and the leading haptic is externalized using Carlevale forceps. The lens foot process will sit in the scleral grove that has been created. The same is done for the trailing haptic via a handshake using a 23 Eckardt's forceps. The Eckardt forceps are used to feed the haptic to the Carlevale forceps and should not be used for the externalisation, as they can cut the haptic. The scleral incision is sutured with 8–0 Vicryl; the suture should not catch the lens foot process with the aim to cover the haptics using the anterior lips of the sclera. The conjunctiva is sutured with the same suture, and this is repeated on the other side. In our experience the haptics do not extrude and have had good safety and refractive outcomes for more than 1 year of follow up data. We hope you will consider this technique as it allows faster lens fixation without post-operative ocular hypotony. Thank you for watching this video. Please email us if you have any questions.

## Declaration of competing interest

The authors declare the following financial interests/personal relationships which may be considered as potential competing interests: 10.13039/100015277Roche Products Ltd. supported with funding for the article submission charges by a hands-off grant. Roche Products Ltd. did not have any involvement in the preparation, drafting, or editing of the manuscript, or in the choice of authors.

